# Overview of the Complex Figure Test and Its Clinical Application in Neuropsychiatric Disorders, Including Copying and Recall

**DOI:** 10.3389/fneur.2021.680474

**Published:** 2021-08-31

**Authors:** Xiaonan Zhang, Liangliang Lv, Guowen Min, Qiuyan Wang, Yarong Zhao, Yang Li

**Affiliations:** Department of Neurology, First Hospital of Shanxi Medical University, Taiyuan, China

**Keywords:** complex figure test, visuospatial ability, digital scoring method, neuropsychological test, neuropsychiatric disorders

## Abstract

The Rey–Osterrieth Complex Figure (ROCF) test is a commonly used neuropsychological assessment tool. It is widely used to assess the visuo-constructional ability and visual memory of neuropsychiatric disorders, including copying and recall tests. By drawing the complex figure, the functional decline of a patient in multiple cognitive dimensions can be assessed, including attention and concentration, fine-motor coordination, visuospatial perception, non-verbal memory, planning and organization, and spatial orientation. This review first describes the different versions and scoring methods of ROCF. It then reviews the application of ROCF in the assessment of visuo-constructional ability in patients with dementia, other brain diseases, and psychiatric disorders. Finally, based on the scoring method of the digital system, future research hopes to develop a new digital ROCF scoring method combined with machine learning algorithms to standardize clinical practice and explore the characteristic neuropsychological structure information of different disorders.

## Introduction

**Figure 1:**
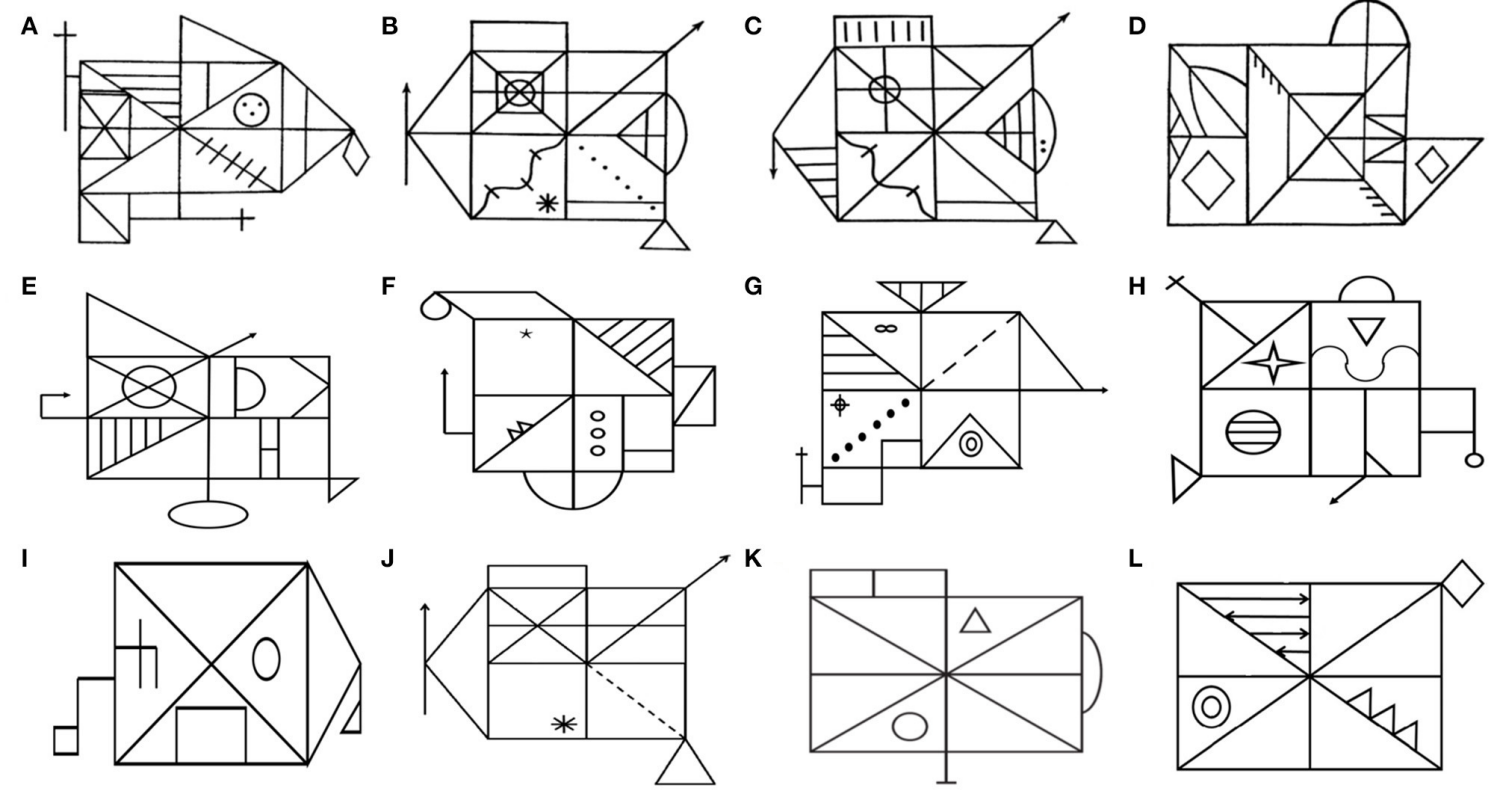
Equivalent and simplified versions of Rey–Osterrieth Complex Figure (ROCF). In 1941, Rey developed the classic ROCF **(A)**. To avoid the learning effect by using the same figure twice, some studies have developed multiple equivalent versions of the ROCF, such as the Taylor figure **(B)**, the modified Taylor figure **(C)**, the Mark figure **(D)**, and various versions of the Medical College of Georgia Complex Figures **(E–H)**. Subsequently, multiple simplified versions of ROCF **(I, K, L)** were developed. The Benson figure **(I)** is less affected by executive function. **(K)** has been proven to be suitable for the elderly. Kim developed **(L)** for digital assessment tools. Similarly, the simplified Taylor figure **(J)** has also been confirmed to suit the low-educated elderly.

The Complex Figure Test was first designed by (1) in 1941 and then standardized by (2) in 1944, which provided preliminary standardized data for 230 children and 60 adults to form the widely used Rey–Osterrieth Complex Figure (ROCF) test (Figure 1A). The ROCF is mainly used to evaluate visuo-constructional ability and non-verbal memory in clinical practice and research. It includes immediate copy and delayed recall. Subjects copy complex geometric shapes and then reproduce them from memory. The neuropsychological dysfunction of a subject can be assessed by drawing performance, including attention and concentration, fine-motor coordination, visuospatial perception, non-verbal memory, planning and organization, and spatial orientation (3, 4). Graphomotor impressions are a product of complex cognition, perception, and motor skills (5). Moreover, graphics rendering makes the task more complicated, which involves organizing graphics into meaningful perceptual units. Therefore, ROCF is widely used to study the neural relevance of structural functions in healthy individuals and the mapping disorders of patients with dementia and other brain diseases (6–10).

## Versions of Complex Figure Test

### Equivalent Versions

At present, multiple equivalent and simplified versions of ROCF have been developed for different research purposes. The ROCF was initially used as a tool to assess the visuospatial abilities of brain injury, such as before and after the evaluation of the efficacy of brain trauma surgery, medication, and rehabilitation (11, 12). To avoid the learning effect of using the same figure twice, some studies have tried to develop multiple equivalent versions of the ROCF. In 1969 (13), developed a complex figure (Taylor figure; Figure 1B) as a postoperative memory test to study the influence of left and right temporal lobe resection on non-verbal task recall and recognition test, while the ROCF is a preoperative memory test. However, some studies have found that the Taylor figure is easier to organize and learn than the ROCF and is easier to remember in the recall. Tombaugh and Hubley (14) found that they produced similar scores in copying, but the Taylor figure had higher scores in recall. He further analyzed the differences in recall and interpreted it as the Taylor figure being easier to describe and memory-encode.

To solve this problem, Hubley reduced some distinct parts of the Taylor figure, added some lines to increase the complexity of the visual test, and adjusted the position of characteristic details. A subsequent verification of the equivalence between the modified Taylor figure (Figure 1C) and the ROCF shows that they have the same complexity in evaluating visual–spatial construction and visual memory (15). Some researchers have also developed the Mark figure (Figure 1D) (16) and various versions of the Medical College of Georgia Complex Figures (Figures 1E–H) (17, 18) and found that there was no significant difference between them and the ROCF in the copy and recall scores. It indicated that they are comparable and can be used interchangeably in clinical practice.

### Simplified Versions

With the use of the ROCF and its equivalent versions in clinical practice, its shortcomings have gradually emerged (7, 19–21). First, the standard of ROCF copying and recall of the elderly is very low. Twenty-seven points (total score of 36 points) is considered the expected standard, which is in the first percentile among 55–59 years old (19). Second, with the improvement of education level, the scores of immediate copy, delayed recall, and recognition of ROCF increased significantly (7, 21). Therefore, the educational level should be stratified when formulating the cutoff value of the scale. Third, when the mapping task is used for low-educated people (illiterate), the ability to distinguish between patients and non-patients will be lost (20). One should also be careful of the interpretation of the results of complex drawing tasks, even if the level of education is stratified. Some studies have developed multiple simplified versions of the ROCF to solve the above-mentioned problems. Poreh et al. (22) developed a simplified ROCF (Figure 1K) and found that its copying, recall, and strategy scores are well-distributed among healthy older people (over 60 years old), indicating that it is more suitable for the elderly than the classical ROCF. Similarly, de Paula et al. (23) developed a simplified Taylor figure (Figure 1J) and verified the validity and the reliability of its assessment of the visuospatial ability of the low-educated elderly (primary school). It was found that the figure had a high internal consistency for the copy (0.89), immediate recall (0.97), and delayed recall (0.96) components, providing evidence of test reliability, and had a significant correlation with Stick Design Test and DRS Constructional Praxis. It was suitable for the low-educated elderly.

Besides this, to reduce the impact of executive function on the ROCF replication performance (24), used a simplified ROCF (Benson figure, developed by Frank Benson, M.D; Figure 1I) to explore the difference in visuospatial impairment between Alzheimer's disease and behavioral variant frontotemporal dementia. It could distinguish them well (24). To also extract the parameters stably and accurately in digital and automated CFT assessment tool development (25), developed a simplified ROCF (Figure 1L). The figure has few strokes and is not easy to overlap in a small space.

In short, researchers can choose different equivalent and simplified versions of the ROCF according to various research objects and purposes and further promote the clinical application of the Complex Figure Test.

**Figure 2:**
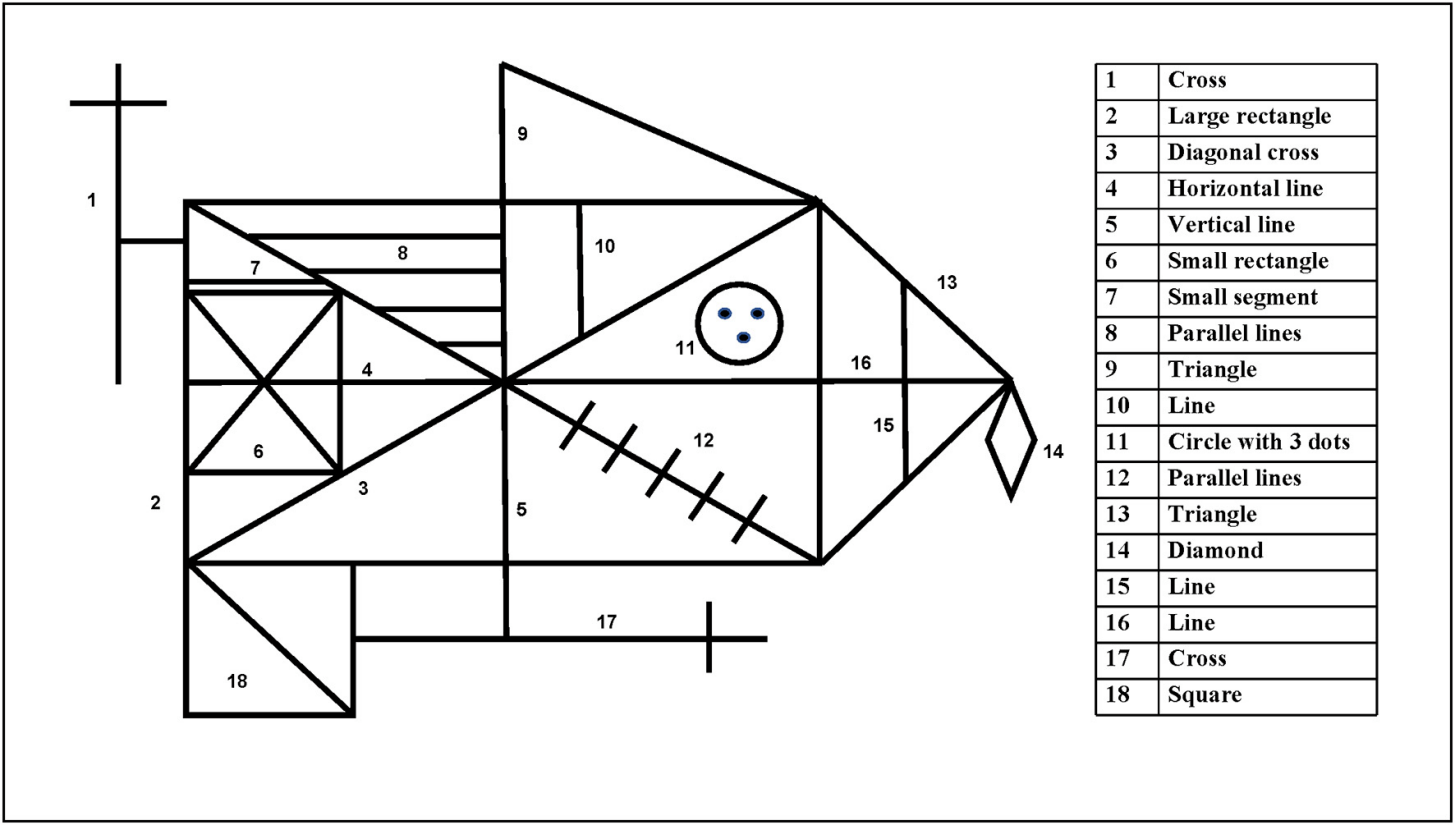
The 18 scoring units of the Rey–Osterrieth Complex Figure.

## Clinical Application of Complex Figure Test

### Traditional Scoring Methods

More than a dozen scoring methods have been developed for the Complex Figure Test (3), which are mainly divided into two categories: the accuracy scoring method and the process scoring method (26). This review here only introduces the scoring methods commonly used in clinical practice and research. The accuracy scoring methods (2, 27–29), are used to quantitatively score the accuracy of each unit and the relative position in the figure, which reflects the matching degree of the drawn figure with the original design presented. It is mainly used to evaluate the visuo-constructional ability and visual memory. The process scoring methods Bennett-Levy (30), Boston Qualitative Scoring System (BQSS) (31), Developmental Scoring System (32), Denman (33), Savage (34), Booth's (35, 36), and Q-score (37, 38) are used to score the direction and order of drawing and the placement of figure units. Thus, they increase executive function and organizational strategy evaluation to evaluate cognitive function from multiple dimensions (26). These scoring methods are applicable to all equivalent and simplified versions of ROCF (see Table 1 for the specific scoring criteria).

**Table 1:** Traditional scoring methods of the complex figure test.

**Scoring methods**	**Variables**	**Features**	**References**
Osterrieth	Accuracy, placement	(1) Divide ROCF into 18 units (Figure 2), simple and subjective(2) With a maximum score of two points for each unit for a total of 36 points. Two points for accurate figure and correct location; one point for accurate figure, incorrect location or inaccurate figure, and correct location; 0.5 point for inaccurate figure but recognizable and incorrect location; 0 point for missing figure that cannot be recognized	(2)
Taylor	Accuracy, placement	(1) Based on the Osterrieth scoring, detailed scoring rules were formulated for the accuracy and location of each unit, and the scoring criteria were further refined	(29)
Meyers	Accuracy, placement	(1) Based on the Osterrieth scoring, the accuracy and location of each unit are clearly defined, and the ambiguity errors of all units are evaluated(2) Add recognition test (24 patterns)(3) Simple and objective	(27, 28)
Bennett-Levy	Continuity, symmetry, and strategy score	(1) Scoring for important continuation points and successive construction of symmetrical units or symmetrical components(2) Symmetry score can be used to assess executive dysfunction	(30)
Developmental Scoring System	Organization, accuracy, errors, and a style rating (part-oriented, intermediate, and configurational)	(1) Suitable for children(2) Refer to the developmental level of visual organization of children(3) Rating for style can provide useful indicators of developmental trends	(32)
Denman	Construction accuracy (line angles, line length, line number, and sector location)	High scores indicate an excellent ability to construct and recall visual details rather than how well the component is organized	(33)
Savage	Construction accuracy (33) and organization strategy Quantitative analysis and descriptive analysis of organizational sequence	(1) Add the assessment of organizational ability based on Denman(2) Descriptive analysis provides information about the early organizational sequence	(34)
Boston Qualitative Scoring System	Presence, Accuracy, Placement, Fragmentation, Planning, Neatness, Size distortion, Rotation, Perseveration, Confabulation, Asymmetry, and six summary scores based on these dimensions (presence and accuracy scores, immediate retention, delayed retention, organization, etc.)	(1) A comprehensive scoring method suitable for adults(2) Mainly used to assess executive dysfunction, and five variables (fragmentation, planning, organization, neatness, and perseveration) can be used to measure executive function	(31)
Booth's	Order index, Style index, and Central Consistency Index(CCI)	Assess the central consistency of eating disorders	(35, 36)
Q-score	Unit score, Order score, and Q-score	Assess the central consistency of eating disorders	(37, 38)

The accuracy scoring method is a single quantitative scoring method. Although it has good clinical practicality and high inter-rater reliability (Osterrieth 99%, Meyers 94%), it cannot assess the organization strategy and executive function of drawing. Therefore, meaningful information about the drawing process will be missed, such as whether the figure is drawn in an organized and planned way or fragmented and chaotic (26). The process scoring method is a comprehensive scoring method that can comprehensively evaluate cognitive function. The executive variables of BQSS can distinguish executive disorders from normal people, including vascular dementia (9), Parkinson's disease (39), schizophrenia (40), attention deficit hyperactivity disorder (41), and senile depression (42), and it is more valuable in exploring poor performance in the recall—for example, a BQSS-based visuospatial memory study found that poor recall performance in schizophrenia is mediated by a defect in the organizational strategy of copying (40). However, due to the shortcomings of process scoring methods, such as time-consuming and standardized training, it is not widely used in clinical practice (31).

### Clinical Application in Neurological Diseases

Neurodegenerative and cerebrovascular diseases can cause cognitive impairment, such as Alzheimer's disease, Lewy body dementia, frontotemporal dementia, Parkinson's disease dementia, and cerebral small vessel disease. The clinical subtypes of mild cognitive impairment (MCI) are the precursor forms of various dementias, and early recognition is of great significance for disease management (43). The ROCF is widely used to assess the visuo-constructional ability and visual memory of brain injury or cognitive disorders.

#### Alzheimer Disease

Alzheimer's disease (AD) is a neurodegenerative disease characterized by progressive cognitive impairment and behavioral disorders. Memory impairment is its core cognitive feature (44). The structure or function of the parietal lobe can be changed in the early stage, so the visuospatial ability test may be more accurate than other tests in distinguishing AD from non-AD (4). Some studies have confirmed that the Complex Figure Test has good diagnostic and prognostic potential for AD (9, 24). de Paula et al. (23) studied the performance of healthy elderly, MCI, and AD in the simplified Taylor figure (Figure 1J). It was found that there were statistical differences in the scores of copying, immediate recall, and delayed recall among the three groups, and there was a progressive damage pattern. Moreover, the impairment of the graphic recall test may help describe the characteristics of MCI and AD. At the same time, ROCF copy performance in AD patients is associated with low metabolism in bilateral temporal–parietal, occipital, and right frontal cortical areas (45), especially in the BA40 and BA7 zones (Brodmann zone) (46). It shows that the ROCF seems to reflect the function of the posterior temporal–parietal cortex and emphasizes the role of visuospatial processing in AD.

#### Vascular Dementia

Vascular dementia (VaD) is the second major cause of dementia after AD and can be prevented. Cerebral small vessel disease is a common cause of VaD, and the lesion is located in the subcortical area (47). It can lead to the destruction of the cortical striatal loop and decreased frontal lobe function, resulting in impaired attention and processing speed (48). Therefore, cognitive scale assessment should focus more on cognitive domains such as executive function, attention, and visuospatial ability. VaD and AD have common pathogenic factors and pathological mechanisms; especially when AD patients are mixed with vascular factors, clinical identification is more complex (4). Previous studies have found that patients with subcortical vascular mild cognitive impairment (v-MCI) performed poorly in ROCF copying (49). Salvadori et al. (9) used the Boston Qualitative Scoring System to explore the reasons further and found that v-MCI patients show more planning and organizational deficits than aMCI patients. Their reproductions are more fragmented and contain more perseverations. Therefore, the Complex Figure Test can distinguish them well.

#### Parkinson's Disease and Lewy Body Dementia

Parkinson's disease (PD) is a common neurodegenerative disease with complex clinical symptoms, including motor symptoms such as retardation, muscle rigidity, and resting tremor, non-motor symptoms such as hyposmia, constipation, and rapid eye movement sleep, behavior impairment and cognitive impairment, etc. (50). Cognitive impairment (PD-MCI) is more common, and 70–80% of PD patients can progress to dementia with severe executive dysfunction. Scarpina et al. (39) found that the Boston Qualitative Scoring System's copy total score can distinguish PD patients from normal people. The low planning and neatness scores of PD patients indicate that poor copying performance is related to executive function, especially planning and impulsivity, rather than impaired visuo-constructional ability (39). Since the subthalamic nucleus plays a vital role in optimal planning, planning defects are related to the dysfunction of the frontal basal ganglia network (regulatory response inhibition) (51). PD patients also performed poorly on the ROCF delayed recall, which was explained as memory retrieval failure due to the destruction of the frontostriatal loop (52). Visuospatial impairment is also an early feature of PD-MCI, and figure copying tests have been shown to predict the progressive cognitive decline of PD, such as pentagons and cubes (53). The ROCF also has good screening and diagnostic capabilities for PD-MCI and can be used as a predictive factor (54).

Lewy body dementia (DLB) is the second most common neurodegenerative dementia after AD, and its main manifestations are memory impairment, Parkinson's syndrome, and hallucinations. It has the same pathophysiological mechanism as PD and belongs to alpha- synucleinopathy (55). The diagnosis of typical PDD and DLB is based on the order of appearance of dementia and Parkinson's symptoms, but it is difficult to distinguish in clinical practice. This is because their symptoms overlap and are atypical. However, studies have found that DLB and PDD have different cognitive characteristics (56). DLB has more severe cognitive impairment than PDD, especially attention, visuospatial ability, and language. It is explained that they have different AD co-pathological levels (different β-amyloid content and location; PD <10%, PDD <40%, and DLB >70%), and the ROCF copying test can distinguish them well.

#### Frontotemporal Dementia

Frontotemporal dementia (FTLD) is a type of dementia with damage to the frontal and temporal lobes, which is clinically characterized by behavioral and language disorders (44). Previous studies on ROCF in AD and FTLD have been controversial (4, 57), which may be caused by different neuroanatomical bases and cognitive mechanisms. Poor copy performance in AD is significantly related to atrophy of the right parietal cortex, involving spatial perception and attention. In contrast, poor performance in FTLD is related to atrophy of the right dorsolateral prefrontal cortex, involving spatial planning and working memory. Based on the above-mentioned findings, (24), proposed an alternative evaluation method. He uses a simplified ROCF (Figure 1I) less affected by executive functions to evaluate visuospatial ability, which can better distinguish them. In addition, the delayed recall of ROCF can also distinguish them well (44).

#### Cerebellar Ataxia and Amyotrophic Lateral Sclerosis

The cerebellum not only coordinates movement but also involves cognitive processes, including visuospatial abilities. Cerebellar injury shows poor visuospatial skills. Resting-state functional connectivity studies show the connection between the cerebellum and the parietal lobe involved in visuospatial integration (58). The cerebellum may contribute to figuring organization to promote effective visuospatial integration and perception while encoding shapes. Slapik et al. (10) found that ROCF copying of cerebellar ataxia was poor, but the subsequent recall was normal, and recall accuracy is related to processing speed. It shows that copying strategy compensates for recall performance, and organizational strategy can affect ROCF performance more than visuospatial skills. The ROCF is also used to assess visuospatial memory in amyotrophic lateral sclerosis (59). The prefrontal lobe injury can appear in the early stage and manifests as reduced executive function. The more severe the executive dysfunction, the worse the ROCF performance. Some patients even meet the diagnostic criteria of frontotemporal dementia, which has been proved to be related to the destruction of the visual cortex striatal circuit and the prefrontal lobe circuit (60).

#### Traumatic Brain Injury and Brain Tumors

Mild traumatic brain injury (mTBI) can cause various cognitive, behavioral, physical, or emotional symptoms. The study found that, after the expected recovery period of 2–12 weeks, 10–20% of mTBI patients still experience persistent post-concussion symptoms, significantly impacting their quality of life and social integration (61). Visual and verbal memory processes are the two most severely impaired cognitive functions after mTBI (62). The ROCF helps distinguish different visual memory processes (including immediate recall, delayed recall, and recognition). L'Ecuyer-Giguere et al. (63) found that mTBI patients have short- and long-term visual memory impairment when drawing ROCF, while visual recognition function is relatively retained. Both verbal and performance IQ seem to be related to visual memory performance. It is explained that recall depends on the hippocampus, and familiarity depends on the medial temporal cortex. In addition, ROCF (or its equivalent) is also used to assess constructional apraxia in elderly patients with focal brain tumors (benign or malignant), which are common in parietal and parieto-occipital lesions (64). Constructional apraxia is a neuropsychological syndrome defined as the apparent impairment of the ability to build, assemble, or draw two- or three-dimensional models (simplex or complex), synthesizing its individual elements into a complete object. This task requires the analysis and reproduction of the spatial relationship between individual elements under the guidance of perceptual or mental models, which involve different areas of the brain (e.g., occipital lobe, parietal lobe, and frontal lobe) (65). ROCF, which involves multiple cognitive domains, is considered the most reliable test for determining constructional apraxia. Retrospective literature shows that focal brain tumors might cause overall cognitive deficits. ROCF may be affected by this general injury, and there is no specific correlation with the side and location of the lesion, making the processing task too difficult (64).

### Clinical Application in Psychiatric Diseases

The ROCF has also been used to evaluate visuospatial abilities, learning, visual memory, and executive dysfunction in some psychiatric diseases, including attention deficit hyperactivity disorder, eating disorder, and schizophrenia.

#### Attention Deficit Hyperactivity Disorder

Attention deficit hyperactivity disorder (ADHD) is a neurodevelopmental disorder of children and adolescents. It has cognitive deficits in the dorsolateral prefrontal cortex that regulate executive functions, manifested by decreased working memory and attention (66). Hyun et al. (41) used digital ROCF to evaluate the visuospatial working memory of adolescents with ADHD. He found that the deviation value (the pixel difference between the original image and the template image) of ADHD was different from that of the control group and was negatively correlated with the visuospatial index and the working memory index. However, there was no difference in the deviation of immediate recall between the two groups, indicating no memory storage defect in ADHD patients. The ROCF has also been gradually used in the executive function training of children with ADHD. After training, the performance of ADHD patients in executive function tasks can be comparable to that of normal children (67). ROCF is a reliable measure to evaluate the executive function of children and adolescents (68).

#### Eating Disorder

The ROCF was also used to assess cognitive deficits in eating disorders (anorexia nervosa and bulimia nervosa), such as executive function, visuospatial ability, learning, memory, language, and attention. In recent years, studies have shown that they are related to weak center consistency (69). The weak center consistency hypothesis is a cognitive theory that refers to a processing style that focuses on details rather than the overall, which is common in patients with anorexia nervosa. Lang et al. (36) found that the center consistency index based on Booth's scoring can be better used to assess the weak center consistency of eating disorders. However, it pays too much attention to drawing element order, ignoring the overall figure, and the scoring method is too complicated. Subsequently, Weider et al. (38) used the Q-score to assess the weak central consistency of eating disorders and found that it has visual construction and visual memory defects, and the Q-score was strongly correlated with the center consistency index. Thus, it shows that the Q-score is suitable for measuring the weak center consistency of eating disorders.

#### Schizophrenia

Initially, Göder et al. (70) found that patients with schizophrenia performed poorly on the ROCF recall, which is consistent with the view of temporal lobe dysfunction. It will affect the connection between external stimuli and semantic meaning and then affect the visual–spatial memory ability. Subsequently, Kim et al. (40) used the Boston Qualitative Scoring System for further research and found that there were significant differences in some scoring variables (e.g., fragmentation in copying; configuration presence and planning in immediate recall; configuration presence, combination presence, detail presence, fragmentation, planning, and neatness in delayed recall) between the schizophrenia and control groups, and the organization strategy of copying mediates the difference between the two groups in immediate recall. It shows that organizational deficits seem to be related to visual memory deficits in schizophrenia. The ROCF has also been used to measure the learning potential of schizophrenia, which is defined as the ability to acquire cognitive skills and use them to solve problems under appropriate circumstances (71). The organizational strategy of ROCF copying can give it intensive guidance.

## Digital Complex Figure Test

Dementia or other brain disease patients show different drawing behavior patterns (5)—for example, AD patients have a poor ability to integrate details and break the figure into fragmented parts instead of focusing on the overall structure. There is an increased tendency to exhibit abnormal behaviors when selecting drawing areas, organizing graphics, determining drawing order, and other activities. However, the traditional scoring methods based on pen and paper have limitations for detecting these abnormal behaviors. Therefore, it is necessary to establish a scoring method for a digital device to increase the scoring of the drawing process (25).

**Figure 3:**
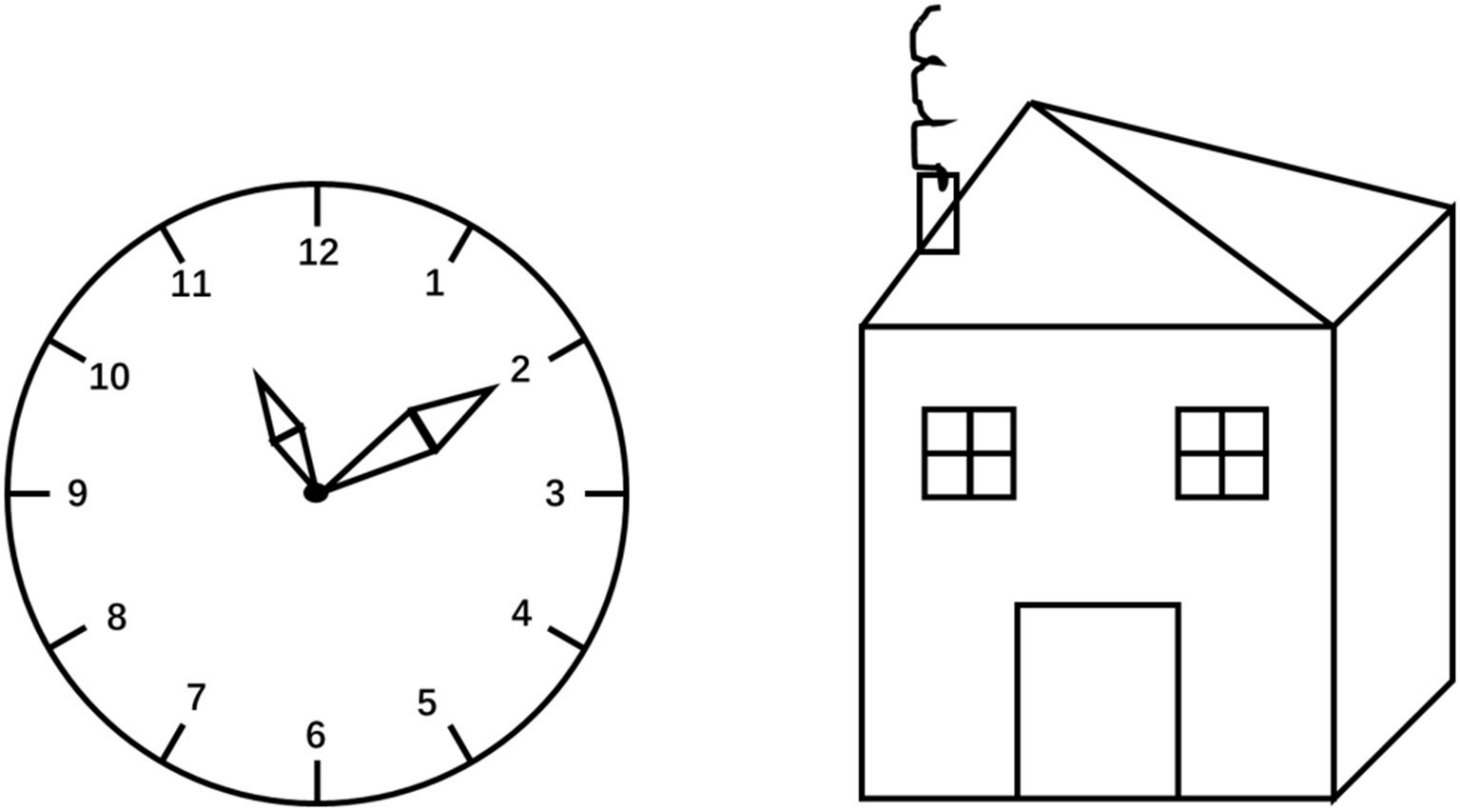
Clock and house drawing tasks.

With the rapid development of technology, the digitization of traditional cognitive scales has become a hot spot in neuroscience. The digital clock drawing test (Figure 3) is relatively mature. Some studies use digital devices (Anoto Inc digital pen or Windows Surface Pro 4 tablet) to collect the physical parameters of drawing, including kinetics (time in air, time on surface, total time, drawing speed, etc.) and device–human interaction values (pressure, pressure/velocity relation, strokes per minute, pen-up stroke length, etc.) (72–74). This method can more accurately reflect the drawing processing speed, motion control ability, and organization strategy and increase the understanding of the drawing strategy and executive function of the subject. Studies have shown that the digital clock drawing test can identify cognitive impairment with Alzheimer's disease (73), perioperative period (75), and Parkinson's disease (76). A recent study used the neural network employing information–theoretic feature selection approaches to perform machine learning analysis on the features collected by the digital clock drawing test. The results show that the classification accuracy between the two groups of different subtypes of MCI and AD is as high as 83.69% (43). Furthermore, it shows that applying machine learning technology to neuropsychological tests is expected to be an effective first-line screening method for classifying different types of cognitive impairment. This technique can measure subtle and discrete behaviors, including drawing delays, thinking decisions, and graphical motion output, which can better distinguish subtypes of dementia.

Since the drawing of a clock, a house (Figure 3), and a tree involves ventral visual pathways that will elicit semantic knowledge, the use of abstract ROCF may be more objective. In addition, ROCF includes the recall test that requires visuospatial working memory before it can be translated into motor skills and executive plans, involving dorsal visual pathways (45). Therefore, the Digital Complex Figure Test has better application prospects.

Initially, Canham et al. (77) used computer vision technology to scan the ROCF offline and located the 18 units of the Osterrieth scoring. The results show that the positioning and perception grading of geometric features (large rectangle, triangle, and diamond) have a high success rate. Similarly, Hyun et al. (41) used a new Gaussian filter method to extract ROCF drawn on the tablet and analyzed the difference by comparing the pixel difference between filtered template images and filtered original images. It can be used to assess different patterns of visuospatial working memory in attention deficit hyperactivity disorder. Neither of these studies evaluated the drawing order but focused on identifying outlines and details related to traditional scoring methods.

Subsequently, Poreh et al. (22) used a new technology (digital pen) to capture the drawing process and recorded the movement of the pen to the laptop through an infrared receiver. It realized the semi-automatic analysis of Bennett-Levy scoring and found that the symmetry score can be a good marker for non-verbal executive function. To finely analyze drawing behavior patterns and establish a simple and objective digital scoring method, Kim et al. (25) used a tablet (Samsung Galaxy Book 12) to record the drawing process and automatically extract stroke parameters (time, speed, and length) and graphical space information (position of center and mass). It also uses 2D technology to analyze the shape similarity between the original and copied figures. The results found that AD patients copied the figure in a more fragmented way with a longer pause and were more inclined to move the figure closer to the target image with lower accuracy than normal control. Late-onset AD showed signs of leftward deviation when drawing the figure (25). The digital Complex Figure Test can quantitatively evaluate drawing performance and further clarify organizational strategies. The number of long strokes and the speed of the longest stroke can reflect the execution function because they are most likely to be used to build the skeleton of the figure. The position of the figure reflects the ability of spatial arrangement and can prompt signs of neglect and proximity on one side of the space.

## Conclusion and Prospect

The ROCF is a comprehensive neuropsychological assessment tool involving multiple cognitive domains and is less affected by language and culture. At present, a variety of versions and scoring methods have been developed, and researchers can choose according to different clinical and research purposes. ROCF can study the cognitive deficits in patients with dementia and other brain diseases and further explore the reasons for poor performance in copying and recall. Especially the development of digital Complex Figure Test can quantitatively evaluate the drawing process and further clarify the drawing strategy of the patient by comparing the trajectory of the pen tip, the spatial arrangement, and the similarity of the completed drawing. Inspired by digital clock drawing technology, digital equipment can collect more physical parameters, such as drawing dynamics and device–human interaction, and then more refined analysis of drawing processing speed, motion control ability, and organization strategy. At the same time, combined with machine learning algorithms, it can build classification and diagnosis models of different diseases. A recent study used the deep learning algorithm to automate ROCF scoring, which has high performance and is close to the reliability of human raters (78). Future research should use machine learning technology to provide information about the neuropsychological structure of different neuropsychiatric diseases and gradually establish a new digital scoring method to standardize clinical practice, such as a digital ROCF feature selection analysis algorithm.

## Author Contributions

YL developed the study concept and the study design. GM, QW, and YZ performed the literature review and produced figures and tables. XZ, LL, GM, QW, and YL wrote the manuscript. All authors contributed to the article and approved it for publication.

## Conflict of Interest

The authors declare that the research was conducted in the absence of any commercial or financial relationships that could be construed as a potential conflict of interest.

## Publisher's Note

All claims expressed in this article are solely those of the authors and do not necessarily represent those of their affiliated organizations, or those of the publisher, the editors and the reviewers. Any product that may be evaluated in this article, or claim that may be made by its manufacturer, is not guaranteed or endorsed by the publisher.
